# Anti-Drug Antibodies in Pigtailed Macaques Receiving HIV Broadly Neutralising Antibody PGT121

**DOI:** 10.3389/fimmu.2021.749891

**Published:** 2021-11-11

**Authors:** Wen Shi Lee, Arnold Reynaldi, Thakshila Amarasena, Miles P. Davenport, Matthew S. Parsons, Stephen J. Kent

**Affiliations:** ^1^ Department of Microbiology and Immunology, The Peter Doherty Institute for Infection and Immunity, University of Melbourne, Melbourne, VIC, Australia; ^2^ Kirby Institute, University of New South Wales, Kensington, NSW, Australia; ^3^ Department of Pathology and Laboratory Medicine, School of Medicine, Emory University, Atlanta, GA, United States; ^4^ Yerkes National Primate Research Center, Emory University, Atlanta, GA, United States; ^5^ Melbourne Sexual Health Centre and Department of Infectious Diseases, Central Clinical School, Monash University, Melbourne, VIC, Australia

**Keywords:** HIV, broadly neutralizing antibodies (bNAb), PGT121, anti-drug antibodies (ADA), pigtailed macaque

## Abstract

Broadly neutralising antibodies (bNAbs) may play an important role in future strategies for HIV control. The development of anti-drug antibody (ADA) responses can reduce the efficacy of passively transferred bNAbs but the impact of ADA is imperfectly understood. We previously showed that therapeutic administration of the anti-HIV bNAb PGT121 (either WT or LALA version) controlled viraemia in pigtailed macaques with ongoing SHIV infection. We now report on 23 macaques that had multiple treatments with PGT121. We found that an increasing number of intravenous doses of PGT121 or human IgG1 isotype control antibodies (2-4 doses) results in anti-PGT121 ADA induction and low plasma concentrations of PGT121. ADA was associated with poor or absent suppression of SHIV viremia. Notably, ADA within macaque plasma recognised another human bNAb 10E8 but did not bind to the variable domains of PGT121, suggesting that ADA were primarily directed against the constant regions of the human antibodies. These findings have implications for the development of preclinical studies examining multiple infusions of human bNAbs.

## Introduction

Monoclonal antibodies to prevent or treat HIV infection are of increasing interest. Passive infusion of bNAbs effectively controls viremia in HIV-infected subjects and SHIV-infected macaques when the strain is sensitive to the bNAb (1–4). Control of HIV with bNAbs will require multiple treatments since the half-life of standard IgG antibodies is commonly 2-3 weeks and the half-life of IgG antibodies with mutations that extend half-lives is commonly 8-12 weeks (5). Repeated treatment of humans with monoclonal antibodies can lead to anti-drug (anti-antibody) antibodies (commonly termed ADA) (6, 7). Anti-HIV bNAbs are commonly heavily mutated away from the germline in the Fab region of the antibody (8), which could result in immunogenic epitopes. A disadvantage of macaque/SHIV studies of bNAbs is the foreign nature of the entire human bNAb (both the Fab and the Fc). Previous work has illustrated that ADA is common when human bNAbs are delivered to rhesus macaques since the entire antibody is foreign and ADA to both the Fab and Fc can occur (9–12).

Although ADA is an important issue in macaque studies of human bNAbs, several knowledge gaps remain. The number of human mAb treatments needed to induce ADA is not well studied. ADA is presumably effectively primed with a limited number of bNAb administrations, then when boosted to high levels by another administration, results in rapid clearance of the bNAb and consequent reduced antiviral efficacy. However, these precise relationships have not been widely studied.

PGT121 is a potent bNAb that binds to V3 glycans of HIV-1 Env and is effective against a majority of HIV-1 strains. PGT121 has been of great interest in cure-related HIV trials, particularly in combination with a TLR7 agonist where partial control of SHIV in the absence of ART was observed (13). HIV cure studies are likely to need multiple bNAb administrations and robust evaluation in macaque pre-clinical models to delineate a precise role. However, PGT121 has been reported to induce ADA in rhesus macaques (*Macaca mulatta*) by both subcutaneous administration and through delivery *via* an adeno-associated vector (10, 14). Although SIV- or SHIV-infected pigtailed macaques (*Macaca nemestrina*) are an important and useful model of HIV-1, ADA to bNAbs in pigtailed macaques has not previously been studied. We analyzed ADA to the bNAb PGT121 in 23 SHIV-infected pigtailed macaques, assessing the frequency and specificity of ADA generated to PGT121 and the relationship of ADA to loss of potency in controlling viremia. This work will help inform future studies of bNAbs in pigtailed macaques.

## Materials and Methods

### Non-Human Primates

Juvenile pigtailed macaques were sourced from the Monash University Animal Research Platform, the Australian National macaque breeding facility. The Monash University and Australian Commonwealth Scientific and Industrial Research Organization Animal Health Animal Ethics Committees approved all macaque studies. The macaques described here were from three separate studies to assess (i) the efficacy of PGT121 in preventing cell-free or cell-associated SHIV_SF162P3_ infection (3), (ii) the efficacy of WT or LALA PGT121 in preventing cell-associated SHIV_SF162P3_ infection and treating ongoing SHIV_SF162P3_ infection (15) and (iii) the efficacy of PGT121 (16) or eCD4-Ig (unpublished) in preventing intrarectal SHIV_SF162P3_ infection in the presence of seminal plasma. The number and type of human antibody exposures for all macaques in this study are listed in **Supplementary Table 1**.

PGT121 WT, PGT121 LALA, eCD4-Ig and the human IgG1 isotype control antibody were all administered intravenously at 1mg/kg one hour prior to challenge with SHIV_SF162P3_. Both PGT121 WT and LALA were purchased from the Center for Antibody Development and Production (Scripps Research Institute) while the human IgG1 isotype control antibody (clone 52H5/TT1204) was provided by Keith Reimann (NIH Nonhuman Primate Reagent Resource). eCD4-Ig was kindly provided by Stuart Turville (Kirby Institute, University of New South Wales) and Michael Farzan (Scripps Research Institute). A pool of human seminal plasma was generated using samples obtained from the Opposites Attract cohort study (17).

### Viral Load Quantification

Viral RNA in the plasma of SHIV_SF162P3_-infected macaques was measured by digital droplet PCR (ddPCR) as described previously (15). The decay rates of plasma viral loads were estimated using an ordinary linear regression method on the log-transformed values of the measurements using GraphPad Prism software.

### ELISA to Measure ADA in Macaque Plasma

ELISAs were performed to measure the level of anti-drug antibodies against full-length PGT121, PGT121 scFv (Creative Biolabs) and 10E8 (NIH AIDS Reagent Program) in macaque plasma. 96-well Maxisorp plates (Thermo Fisher) were coated overnight at 4°C with 1µg/ml of PGT121, PGT121 scFv or 10E8 in PBS. After blocking with PBS containing 4% bovine serum albumin (BSA) and 0.1% Tween-20, duplicate wells of macaque plasma (1:100 dilution in PBS with 0.2% Tween-20, 0.1% BSA and 0.5% NP-40) were added and incubated for 1.5 hrs at 37°C. Next, plates were incubated with a HRP-conjugated secondary antibody specific for macaque IgG (clone 1B3, Kerafast; 1:16,000 dilution) for 1 hr at 37°C. Plates were then developed with TMB substrate (Sigma), stopped with 0.16M sulphuric acid and read at 450nm using the FLUOstar Omega microplate reader. The absorbance values (OD_450_) of macaque plasma samples were background subtracted with wells containing only PBS and normalised to a positive plasma control for ADA (sample from macaque NM08) by dividing the OD values of test samples with the OD values of the positive control. To validate the ELISA, we measured plasma endpoint titres for ADA and found that ADA measured at a 1:100 plasma dilution (normalised OD_450_) correlated strongly with ADA measured by endpoint titre (r = 0.91, p = 0.0001; spearman correlation).

### ELISA to Measure Plasma Concentration of PGT121

96-well Maxisorp plates (Thermo Fisher) were coated overnight at 4°C with 1µg/ml of HIV_BaL_ gp120 (NIH AIDS Reagent Program). After blocking with PBS containing 4% BSA and 0.1% Tween-20, macaque plasma (1:50 and 1:250 dilutions in PBS with 0.2% Tween-20, 0.1% BSA and 0.5% NP-40) was added and incubated for 1.5 hrs at 37°C. Next, plates were incubated with a HRP-conjugated anti-human IgG secondary antibody that does not cross-react with macaque IgG (#2049-05, Southern Biotech; 1:8,000 dilution) for 1 hr at 37°C. Plates were then developed with TMB substrate (Sigma), stopped with 0.16M sulphuric acid and read at 450nm using the FLUOstar Omega microplate reader. Serial dilutions of PGT121 were included on each plate to construct a standard curve, from which the concentration of PGT121 within macaque plasma was calculated using non-linear regression analysis (using the “Hyperbola (X is concentration)” option in GraphPad Prism).

### Statistics

Statistical analyses were performed with Graphpad Prism 8. The correlation of ADA and viral load decay rate was assessed using the non-parametric Spearman test.

## Results

### Induction of Anti-PGT121 Antibodies in Macaques Following Exposure to Human Antibodies

**Figure 1:**
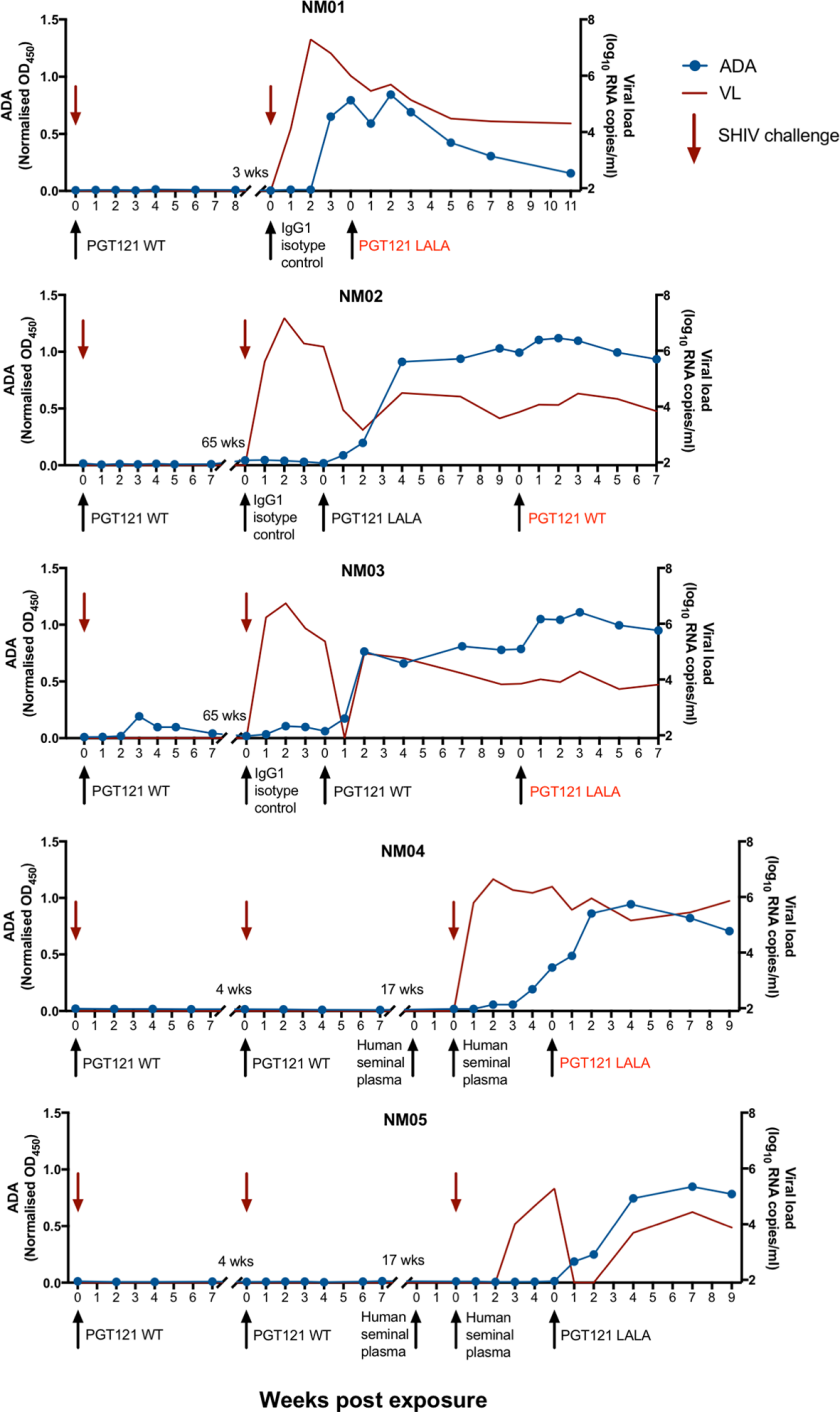
Anti-PGT121 antibodies develop in pigtailed macaques following exposure to human antibodies. Five pigtailed macaques were administered PGT121 or a human IgG1 isotype control antibody intravenously (1mg/kg) prior to challenge with cell-free or cell-associated SHIV. Macaques NM04 and NM05 were administered human seminal plasma either intrarectally or both intravaginally and intrarectally prior to intrarectal challenge with SHIV. Viraemic animals were then infused with PGT121 with either WT or LALA Fc. Macaque anti-PGT121 ADA are shown in the blue line (left y-axis) while SHIV viral loads are shown in the red line (right y-axis). Black arrows indicate antibody administration while red arrows indicate SHIV challenge. The red text indicates failed therapy with PGT121 WT or LALA.

As part of two previous studies (3, 15), a subset of pigtailed macaques were administered either 1mg/kg of PGT121 (with WT Fc) or a human IgG1 isotype control intravenously prior to challenge with cell-free or cell-associated simian HIV (SHIV). Animals receiving PGT121 were protected from viral challenge while animals receiving isotype control developed high levels of viraemia (**Figure 1** and **Supplementary Figure 1**). For a separate study, two animals (NM04 and NM05) were rectally challenged with SHIV following exposure to human seminal plasma and subsequently developed high levels of viraemia (16). The viraemic animals were then used to examine the therapeutic efficacy of PGT121 with either WT Fc or a LALA mutation to abrogate Fcγ receptor engagement (15). While the first therapeutic infusion of PGT121 WT and LALA successfully suppressed viraemia in three of five macaques, two animals (NM01 and NM04) did not exhibit a corresponding decline in plasma viral loads. Viral loads for NM02 and NM03 were suppressed following the first infusion of PGT121 but not after the second infusion. The lack of therapeutic efficacy for PGT121 WT and LALA in certain cases led us to examine whether these five macaques developed anti-PGT121 anti-drug IgG antibodies (ADA) following multiple exposures to antibodies of human origin. As shown in **Figure 1**, all four macaques that failed PGT121 therapy (NM01, NM02, NM03 and NM04) had high levels of PGT121-specific ADA at the time of failed PGT121 therapy (indicated by red text). Most animals developed PGT121 ADA only after 2-3 exposures to either PGT121 or the human IgG1 isotype control antibody. NM03 developed low levels of ADA 3 weeks after the first infusion of PGT121, which waned over time and were boosted to high levels two weeks after the third exposure to a human antibody. Interestingly, while macaque NM04 did not have any ADA following two intravenous infusions of PGT121, ADA were likely primed by the two infusions of human mAbs and the animal developed ADA 4 weeks following two subsequent intrarectal administrations of human seminal plasma (which contains IgG antibodies). PGT121 ADA began to wane in NM01 5 weeks after the last PGT121 exposure but remained high in all other animals.

### Lack of PGT121 Therapeutic Efficacy Due to PGT121-Specific ADA

**Figure 2:**
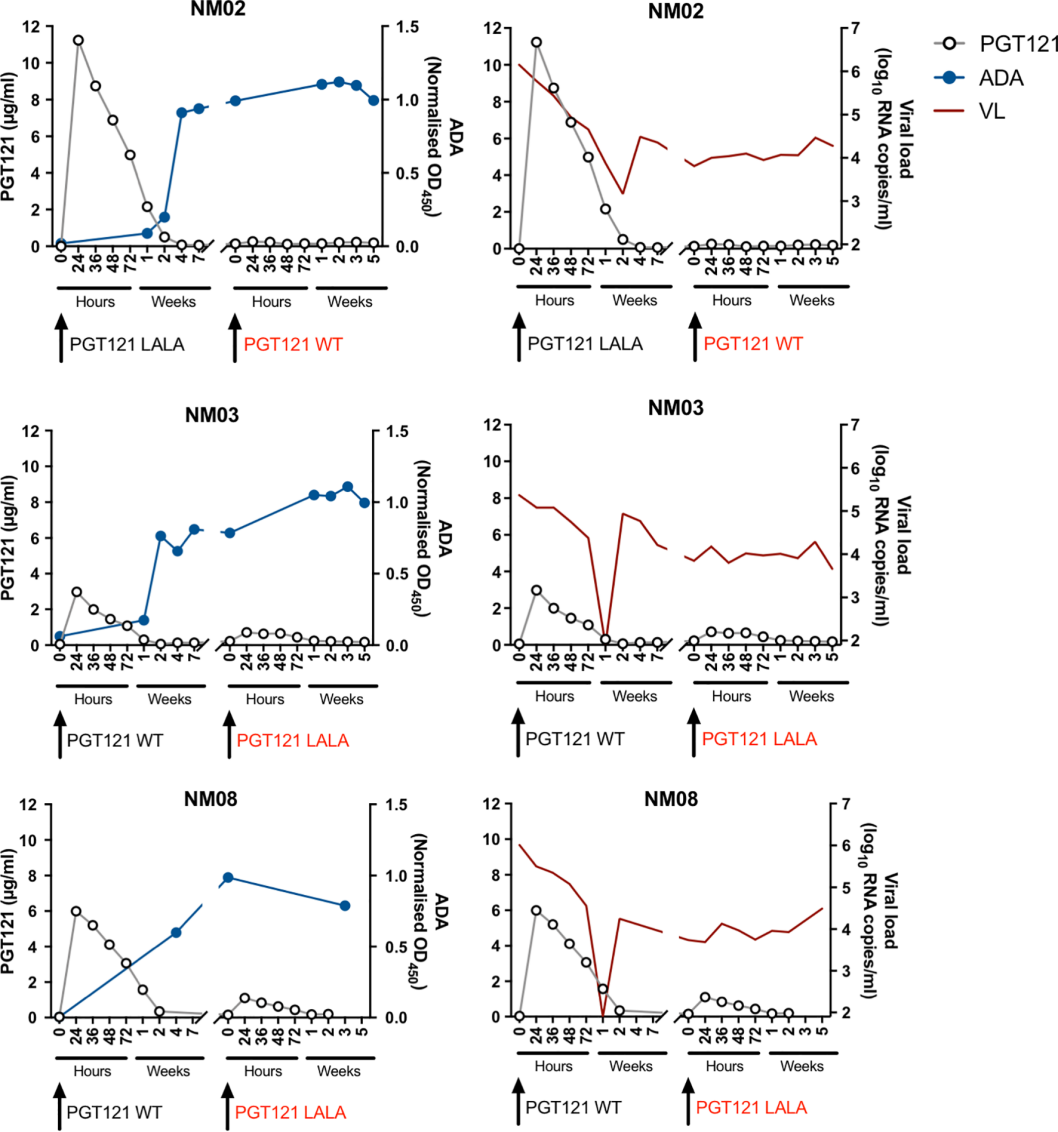
Anti-PGT121 antibodies limit the plasma concentration of PGT121 and diminish the therapeutic efficacy of PGT121 against SHIV. The plasma concentration of PGT121 WT or LALA post-infusion (open circles, left y-axis) is plotted with the level of anti-PGT121 ADA (blue line, right y-axis) or SHIV viral loads (red line, right y-axis). Black arrows indicate antibody administration. The red text indicates failed therapy with PGT121 WT or LALA.

To examine the impact of ADA on the therapeutic efficacy of PGT121 more clearly, we measured the plasma concentration of PGT121 in three macaques that failed PGT121 therapy following the second infusion (**Figure 2**). After the first intravenous infusion, plasma PGT121 levels reached 11.2µg/ml, 3µg/ml and 6µg/ml in NM02, NM03 and NM08 respectively, resulting in a sharp decline in viral loads that eventually rebounded after plasma PGT121 dropped to undetectable levels. In all three animals, PGT121 ADA rose to high levels 2-4 weeks following first infusion of PGT121. ADA remained high at the time of second infusion, resulting in low plasma concentrations of PGT121 and no corresponding decline in plasma viraemia (**Supplementary Figure 2**). These results confirm that the lack of PGT121 therapeutic efficacy was caused by the low levels of bioavailable PGT121 in plasma, likely due to blocking or rapid clearance by PGT121-specific ADA.

**Figure 3:**
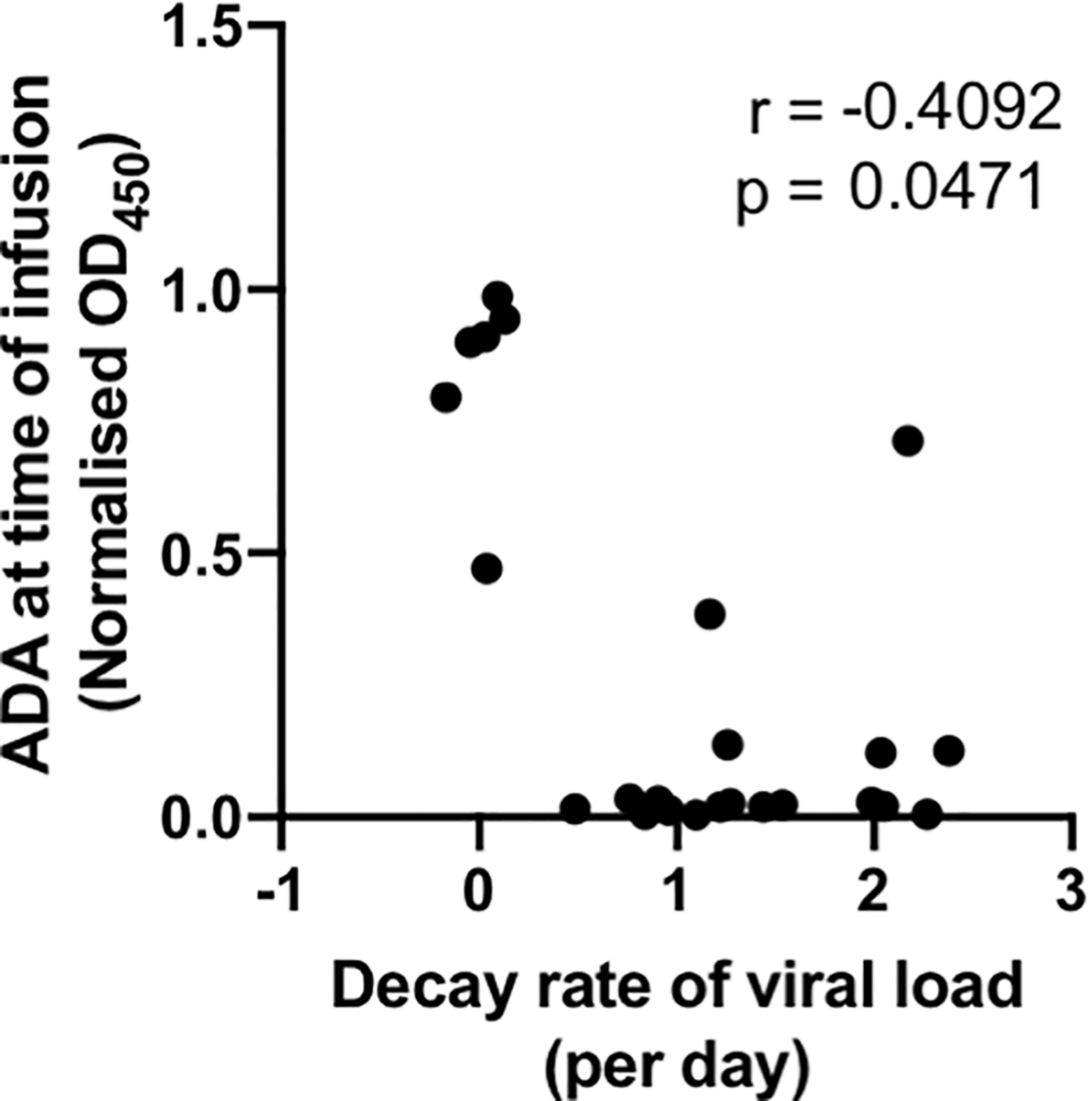
ADA at time of infusion negatively correlates with viral suppression by PGT121. Macaques with active SHIV infection were administered PGT121 WT or LALA. Plasma viral loads were measured by digital droplet PCR at 0, 24, 36, 48 and 72 hours after PGT121 infusion and the decay rate within 72 hours was calculated using linear regression. The correlation was performed using a non-parametric spearman test.

An important measure of the potency of bNAbs and other HIV therapeutics is the rate at which virus decays in the days after administration. With the large number of animals studied, we were able to analyse whether ADA was associated with slower viral decay. We found that the level of PGT121 ADA at the time of infusion negatively correlated with the decay rate of viral loads within 72 hours of PGT121 infusion (r = -0.41, p<0.05; **Figure 3**). A stronger negative correlation between ADA and viral load decay rate is observed if animals without ADA at the time of infusion are excluded (r = -0.62, p<0.05).

### PGT121-Specific ADA Increase With the Number of Exposures to Human Antibodies

**Figure 4:**
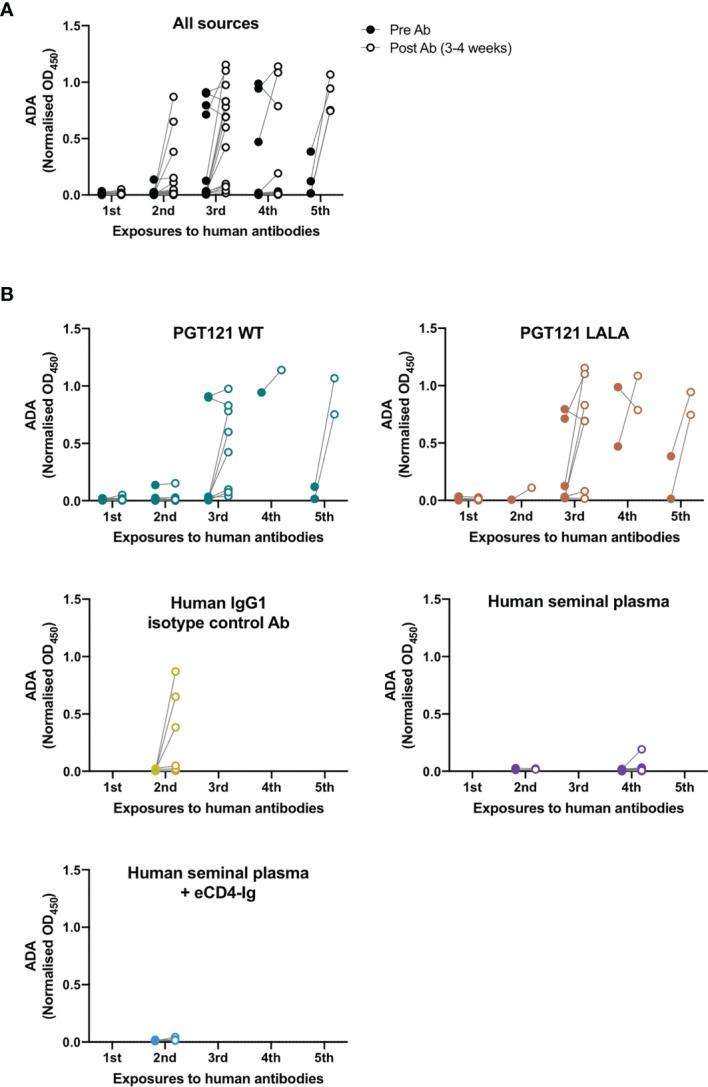
Anti-PGT121 ADA increases with the number of exposures to human antibodies. **(A)** The level of anti-PGT121 ADA in macaques before (closed circles) and 3-4 weeks after (open circles) exposure to human antibodies. **(B)** The level of anti-PGT121 ADA before and after exposure to different sources of human antibodies (intravenous administration of PGT121 WT, PGT121 LALA, eCD4-Ig and human IgG1 isotype control antibody or intrarectal/intravaginal administration of human seminal plasma).

To examine the number of exposures to human antibodies required for the induction of ADA within pigtailed macaques, we measured anti-PGT121 ADA in macaques pre- and 3-4 weeks post-exposure to all sources of human antibodies (**Figure 4A**). Some macaques received two administrations of human seminal plasma (either intrarectally or both intrarectally and intravaginally) two weeks apart (**Supplementary Table 1**), in which case anti-PGT121 ADA was only measured pre- and post-second administration. 23 macaques had been exposed to human antibodies at least twice, with 2 macaques developing very low levels of ADA after first exposure and 4 of 23 macaques developing ADA after second exposure. Following the third exposure, out of 10 macaques that had not seroconverted, a further 7 macaques developed ADA. Interestingly, 7 macaques did not develop ADA following four exposures to human antibodies, while all macaques that had five exposures developed high levels of ADA after the fifth exposure.

We then examined whether the different sources of human antibodies resulted in differential induction of ADA (**Figure 4B**). Intravenous infusions of PGT121 (WT and LALA) and the human IgG1 isotype control antibody all elicited high levels of ADA against PGT121 following 2-5 exposures. Only one animal developed low levels of ADA following intrarectal exposure to human seminal plasma (which contains IgG antibodies) on its fourth exposure to human antibodies. These results show that the elicitation of ADA depends more on the number of exposures to human antibodies rather than the type of antibody exposure.

### ADA Are Specific for the Constant but Not Variable Domains of PGT121

**Figure 5:**
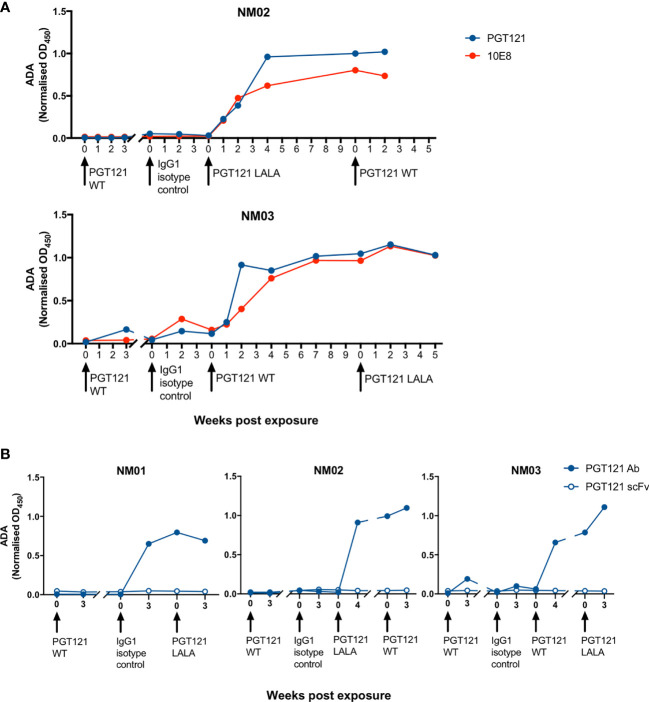
The specificity of ADA responses in pigtailed macaques following multiple infusions of human antibodies. **(A)** Macaque anti-PGT121 and anti-10E8 ADA are shown in blue and red respectively. **(B)** The level of macaque ADA against full length PGT121 (closed circles) and PGT121 scFv (open circles). Black arrows indicate antibody administration.

To interrogate the specificity of ADA within macaque plasma, we next measured the level of antibodies against PGT121 and the gp41-specific bNAb 10E8, which also uses a λ light chain. NM02 and NM03 both had antibodies against PGT121 and 10E8 (**Figure 5A**), implying that the ADA developed from exposure to PGT121 and the isotype control antibody were recognising the constant regions of IgG1 as they were not exclusively specific for PGT121. To confirm these results, we then examined whether macaque ADA could recognise the single chain variable fragment (scFv) of PGT121, which contains only the variable domains of both heavy and light chains (V_H_ and V_L_) without the constant domains (C_H_1, C_H_2, C_H_3 and C_L_). The three macaques tested (NM01, NM02 and NM03) did not have any antibodies recognising the scFv of PGT121 (**Figure 5B**), but had high levels of antibodies recognising full-length PGT121, confirming that the ADA were indeed recognising the constant but not variable domains of PGT121.

## Discussion

The macaque SHIV challenge model of HIV exposure and infection has long been used to examine the protective and therapeutic efficacy of HIV-1 bNAbs isolated from people living with HIV (3, 12, 15, 18–20). bNAbs will generally need to be administered multiple times or delivered continuously *via* viral vectors to maintain efficacy but this poses risks of developing ADA. We describe herein that intravenous administration of human antibodies (1mg/kg) to macaques can lead to the development of anti-PGT121 ADA responses, rising after 2-4 exposures with human antibodies. Interestingly, in an animal that had received two prior infusions of PGT121, anti-PGT121 ADA were detected following two subsequent intrarectal exposures to human seminal plasma (which contains IgG antibodies), showing that mucosal exposure to human antibodies can also lead to the development of ADA. The high levels of anti-PGT121 ADA in these animals resulted in low plasma concentrations of PGT121 following intravenous infusion, impacting the therapeutic efficacy of PGT121 in suppressing SHIV viraemia. The ADA responses did not recognise the variable domains of PGT121 and were therefore not anti-idiotypic, but were directed against the constant domains of PGT121, cross-reacting with a different bNAb, 10E8. While some macaques remained ADA-naïve even after 4 exposures to human antibodies, we recommend that future studies limit the number of infusions of human antibodies to reduce the likelihood of ADA impairing the efficacy of infused antibodies.

The ADA we measured were directed against the constant domains of PGT121 instead of the variable domains. This presumably reflects dominant epitopes in the human IgG1 Fc recognized by the pigtailed macaques, but may also have been exacerbated in our studies as the macaques were exposed to multiple sources of human antibodies (PGT121, human IgG1 isotype control and/or human seminal plasma). The repeated exposures to human IgG1 could have focussed the ADA response to the conserved constant regions instead of the variable domains. A previous study detected PGT121 anti-idiotypic responses in rhesus macaques following two homologous subcutaneous infusions at a higher dose (5mg/kg) (9, 10). A potential way to overcome the elicitation of anti-human ADA in macaques is to “simianise” the bNAbs by grafting the complementarity-determining regions (CDR) of bNAbs onto homologous macaque germline genes with macaque IgG constant regions. While this approach does remove a large portion of immunogenic human antibody epitopes, repeated passive transfer of simianised VRC01 and AAV-delivery of simianised VRC07 still resulted in the development of anti-idiotypic antibodies in macaques (21, 22), reducing the utility of the macaque model in testing repeated or sustained deliveries of HIV bNAbs.

Dosing interval may affect the induction of bNAb-specific ADA, similar to that observed for some human mAbs in use such as TNF inhibitors where longer spaced episodic treatment results in higher induction of ADA compared to regular shorter intervals (23). For our PGT121 studies, we dosed our pigtailed macaques at widely spaced intervals (average 13 weeks, range 2-124) to allow for sufficient drug washout and for SHIV to recrudesce between antibody doses. This wide interval almost uniformly generated ADA after 2-4 doses and thus it was not possible for us to dissect the relative roles of dosing interval or number of doses. One study in rhesus macaques dosed PGT121 at 2 weekly intervals, maintaining control of SHIV, and did not report ADA (13). Future studies of ADA to HIV bNAbs could consider designs to directly assess the role of interval in induction of ADA. Refinement of dosing intervals could be important to limit the generation of ADA responses.

While the elicitation of ADA in non-human primates due to species differences does not translate to humans, bNAbs are typically highly somatically mutated from germline sequences and could potentially be immunogenic. A phase 1 trial of VRC01 administration in humans did not detect anti-VRC01 ADA even after 6 intravenous infusions at 20mg/kg (24). Another phase 1 trial of 3BNC117 and 10-1074 infusion detected ADA in 4 of 18 participants, with one participant having treatment-induced ADA to 3BNC117, one having treatment-induced ADA to 10-1074 and two having anti-3BNC117 ADA at baseline (25). During the study, ADA were not at high enough concentrations to inhibit 3BNC117 or 10-1074 neutralisation of HIV although the ADA, if boosted by further doses of the bNAb, could ultimately limit the effectiveness of the bNAb. Thus, passive infusions of certain bNAbs can result in the generation of ADA responses in humans and should be monitored carefully in future clinical trials.

There is growing interest in utilising bNAbs to prevent or treat HIV infection. Macaque challenge models with SHIV have been a crucial model to test the efficacy of bNAbs preclinically, though the elicitation of ADA responses hampers the ability to examine repeated or sustained deliveries of human antibodies. Our findings suggest that future preclinical studies of human antibodies in pigtailed macaques, similar to rhesus macaques, should limit the number of infusions to avoid generating ADA that can diminish the efficacy of the mAbs of interest.

## Data Availability Statement

The raw data supporting the conclusions of this article will be made available by the authors, without undue reservation.

## Ethics Statement

The animal study was reviewed and approved by the Monash University and Australian Commonwealth Scientific and Industrial Research Organisation Animal Health Animal Ethics Committees.

## Author Contributions

Conceived and designed experiments: WL, MP, and SK. Performed experiments: WL and TA. Analysed the data: WL, AR, TA, and MP. Wrote the paper: WL and SK. Revised the paper: AR, TA, MP, and MD. All authors contributed to the article and approved the submitted version.

## Funding

This work was supported by a National Health and Medical Research Council Program Grant to SK. This work was also supported by the National Institutes of Health’s Office of the Director, Office of Research Infrastructure Programs (P51OD011132). The funders had no role in study design, data collection and analysis, decision to publish, or preparation of the manuscript.

## Conflict of Interest

The authors declare that the research was conducted in the absence of any commercial or financial relationships that could be construed as a potential conflict of interest.

## Publisher’s Note

All claims expressed in this article are solely those of the authors and do not necessarily represent those of their affiliated organizations, or those of the publisher, the editors and the reviewers. Any product that may be evaluated in this article, or claim that may be made by its manufacturer, is not guaranteed or endorsed by the publisher.

## Supplementary Material

Supplementary Figure 1 The development of anti-PGT121 ADA following multiple exposures to human antibodies in individual macaques. Macaque anti-PGT121 ADA are shown in the blue line (left y-axis) while SHIV viral loads are shown in the red line (right y-axis). Black arrows indicate antibody administration while red arrows indicate SHIV challenge. The numbers in red indicate the viral load decay rate (per day) within 72 hours of therapeutic PGT121 WT or PGT121 LALA administration.

Supplementary Figure 2 Mean levels of anti-PGT121 ADA, plasma concentration of PGT121 and corresponding SHIV viral loads in macaques NM02, NM03 and NM08. The mean plasma concentrations of PGT121 WT or LALA post-infusion (open circles, left y-axis) are plotted with the mean levels of anti-PGT121 ADA (blue line, right y-axis) and mean SHIV viral loads (red line, right y-axis). Black arrows indicate antibody administration. The red text indicates failed therapy with PGT121 WT or LALA. The data are depicted as the mean and standard error of the mean.
