# Supplementary material for: Anti-Drug Antibodies in Pigtailed Macaques Receiving HIV Broadly Neutralising Antibody PGT121
Source: Front Immunol. 2021 Nov 11;12:749891. doi: 10.3389/fimmu.2021.749891 (PMC8636046; doi:10.3389/fimmu.2021.749891)
Supplement: Supplementary file 3 [file Table_1.docx]

**Supplementary table 1**: Number and type of human antibody exposure to pigtailed macaques

| **Macaque ID** | **Sex** | **Exposures to human antibodies** | | | | | | | | | |
| --- | --- | --- | --- | --- | --- | --- | --- | --- | --- | --- | --- |
|  |  | **1st** | | **2nd** | | **3rd** | | **4th** | | **5th** | |
|  |  | **Ab** | **Week** | **Ab** | **Week** | **Ab** | **Week** | **Ab** | **Week** | **Ab** | **Week** |
| NM01 | F | PGT121 WT^1^ | 0 | Human IgG1 isotype control^1^ | 11 | PGT121 LALA^1^ | 15 |  |  |  |  |
| NM02 | F | PGT121 WT | 0 | Human IgG1 isotype control | 72 | PGT121 LALA | 76 | PGT121 WT | 87 |  |  |
| NM03 | F | PGT121 WT | 0 | Human IgG1 isotype control | 72 | PGT121 WT | 76 | PGT121 LALA | 87 |  |  |
| NM04 | M | PGT121 WT | 0 | PGT121 WT | 11 | Human seminal plasma (R)^2^ | 33 | Human seminal plasma (R) | 35 | PGT121 LALA | 40 |
| NM05 | F | PGT121 WT | 0 | PGT121 WT | 11 | Human seminal plasma (V/R) | 33 | Human seminal plasma (R) | 35 | PGT121 LALA | 40 |
| NM06 | F | PGT121 LALA | 0 | PGT121 WT | 11 | Human seminal plasma (V/R) | 33 | Human seminal plasma (R) | 35 | PGT121 WT | 40 |
| NM07 | M | PGT121 LALA | 0 | PGT121 WT | 11 | Human seminal plasma (R) | 33 | Human seminal plasma (R) | 35 | PGT121 WT | 40 |
| NM08 | M | PGT121 WT | 0 | Human IgG1 isotype control | 124 | PGT121 WT | 128 | PGT121 LALA | 139 |  |  |
| NM09 | M | PGT121 WT | 0 | PGT121 WT | 11 | Human seminal plasma (R) | 38 | Human seminal plasma (R) | 40 |  |  |
| NM10 | M | PGT121 LALA | 0 | PGT121 WT | 11 | Human seminal plasma (R) | 38 | Human seminal plasma (R) | 40 |  |  |
| NM11 | M | PGT121 LALA | 0 | PGT121 WT | 11 | Human seminal plasma (R) | 38 | Human seminal plasma (R) | 40 |  |  |
| NM12 | M | PGT121 LALA | 0 | PGT121 WT | 11 | Human seminal plasma (R) | 38 | Human seminal plasma (R) | 40 |  |  |
| NM13 | F | PGT121 WT | 0 | PGT121 LALA | 45 | PGT121 LALA | 111 |  |  |  |  |
| NM14 | F | PGT121 WT | 0 | PGT121 WT | 56 | PGT121 LALA | 122 |  |  |  |  |
| NM15 | F | PGT121 WT | 0 | Human IgG1 isotype control | 72 | PGT121 WT | 76 |  |  |  |  |
| NM16 | F | PGT121 WT | 0 | Human IgG1 isotype control | 11 | PGT121 WT | 15 |  |  |  |  |
| NM17 | F | PGT121 LALA | 0 | Human IgG1 isotype control | 11 | PGT121 WT | 15 |  |  |  |  |
| NM18 | F | Human seminal plasma (V/R)^2^ | 0 | Human seminal plasma (R) | 2 | PGT121 LALA | 7 |  |  |  |  |
| NM19 | F | Human seminal plasma (V/R) | 0 | Human seminal plasma (R) | 2 | PGT121 WT | 7 |  |  |  |  |
| NM20 | M | Human seminal plasma (R) | 0 | Human seminal plasma (R) + eCD4-Ig^3^ | 2 | PGT121 LALA^4^ | 10 |  |  |  |  |
| NM21 | F | Human seminal plasma (V/R) | 0 | Human seminal plasma (R) + eCD4-Ig | 2 | PGT121 LALA^4^ | 10 |  |  |  |  |
| NM22 | M | Human seminal plasma (R) | 0 | Human seminal plasma (R) + eCD4-Ig | 2 | PGT121 WT^4^ | 10 |  |  |  |  |
| NM23 | M | Human seminal plasma (R) | 0 | Human seminal plasma (R) + eCD4-Ig | 2 | PGT121 LALA^4^ | 10 |  |  |  |  |

^1^PGT121 WT and LALA and the human IgG1 isotype control antibody were administered intravenously at 1mg/kg for all macaques apart from NM20, NM21, NM22 and NM23

^2^Pooled human seminal plasma (2.5ml) was administered either intrarectally (R) for male macaques or both intravaginally and intrarectally (V/R) for female macaques

^3^eCD4-Ig was administered intravenously at 1mg/kg while pooled human seminal plasma was administered intrarectally

^4^PGT121 WT or LALA were administered at 10mg/kg for macaques NM20, NM21, NM22 and NM23
